# Machine vision-based gait scan method for identifying cognitive impairment in older adults

**DOI:** 10.3389/fnagi.2024.1341227

**Published:** 2024-06-26

**Authors:** Yuzhen Qin, Haowei Zhang, Linbo Qing, Qinghua Liu, Hua Jiang, Shen Xu, Yixin Liu, Xiaohai He

**Affiliations:** ^1^College of Electronics and Information Engineering, Sichuan University, Chengdu, China; ^2^West China School of Medicine, Sichuan University, Chengdu, China; ^3^Department of Geriatrics, Clinical Medical College and Affiliated Hospital of Chengdu University, Chengdu, China; ^4^Department of Endocrinology and Metabolism, National Clinical Research Center for Geriatrics, West China Hospital, Sichuan University, Chengdu, China; ^5^Department of Geriatrics, National Clinical Research Center for Geriatrics, West China Hospital, Sichuan University, Chengdu, China; ^6^Geriatric Health Care and Medical Research Center, Sichuan University, Chengdu, China

**Keywords:** gait, gait recognition, cognitive impairment, machine vision, CNN, BiLSTM

## Abstract

**Objective:**

Early identification of cognitive impairment in older adults could reduce the burden of age-related disabilities. Gait parameters are associated with and predictive of cognitive decline. Although a variety of sensors and machine learning analysis methods have been used in cognitive studies, a deep optimized machine vision-based method for analyzing gait to identify cognitive decline is needed.

**Methods:**

This study used a walking footage dataset of 158 adults named West China Hospital Elderly Gait, which was labelled by performance on the Short Portable Mental Status Questionnaire. We proposed a novel recognition network, Deep Optimized GaitPart (DO-GaitPart), based on silhouette and skeleton gait images. Three improvements were applied: short-term temporal template generator (STTG) in the template generation stage to decrease computational cost and minimize loss of temporal information; depth-wise spatial feature extractor (DSFE) to extract both global and local fine-grained spatial features from gait images; and multi-scale temporal aggregation (MTA), a temporal modeling method based on attention mechanism, to improve the distinguishability of gait patterns.

**Results:**

An ablation test showed that each component of DO-GaitPart was essential. DO-GaitPart excels in backpack walking scene on CASIA-B dataset, outperforming comparison methods, which were GaitSet, GaitPart, MT3D, 3D Local, TransGait, CSTL, GLN, GaitGL and SMPLGait on Gait3D dataset. The proposed machine vision gait feature identification method achieved a receiver operating characteristic/area under the curve (ROCAUC) of 0.876 (0.852–0.900) on the cognitive state classification task.

**Conclusion:**

The proposed method performed well identifying cognitive decline from the gait video datasets, making it a prospective prototype tool in cognitive assessment.

## 1 Introduction

Cognitive impairment, characterized by altered performance in specific cognitive tasks such as orientation, attention, comprehension, memory, reasoning, problem-solving, organizational skills, processing speed, perseverance, and motivation (Allain et al., 2007), can affect multiple domains of cognition simultaneously or consecutively, either gradually or abruptly. Cognitive impairment and dementia are the primary causes of disability in older adults, and promoting healthy brain aging is considered a critical element in reducing the burden of age-related disabilities (Lisko et al., 2021). It is estimated that 40% of dementia might be prevented or delayed by modifying its risk factors, improving activities of daily living (Livingston et al., 2020; Yun and Ryu, 2022). Routine, non-cognitive evaluations alone are insufficient for physicians to accurately predict patients’ cognitive function. Therefore, cognitive assessment facilitates the diagnosis and potential intervention of disorders that impair thinking (Woodford and George, 2007).

The association between motor function and cognition can be understood, in part, in the context of the evolution of human bipedalism (Leisman et al., 2016). Bipedalism served as a significant basis for the evolution of the human neocortex as it is among the most complex and sophisticated of all movements. Gait pattern is no longer regarded as a purely motor task but is considered a complex set of sensorimotor behaviors that are heavily affected by cognitive and affective aspects (Horst et al., 2019). This may partially explain the sensitivity of gait to subtle neuronal dysfunction, and why gait and postural control is associated with global cognitive function in very old people, and can predict the development of disease such as diabetes, dementia, or Parkinson’s disease years before they are diagnosed clinically (Ohlin et al., 2020).

Previous studies reported that slower walking speeds and a greater decline in speed over time are correlated with a greater risk of developing dementia independent of changes in cognition, supporting the role of gait speed as a possible subclinical marker of cognitive impairment (Hackett et al., 2018). Furthermore, spatial, temporal, and spatiotemporal measures of gait and greater variability of gait parameters are associated with and predictive of both global and domain-specific cognitive decline (Savica et al., 2017).

A variety of sensors and machine learning analysis methods have been used in cognitive studies. Chen et al. (2020), for example, used a portable gait analysis system and collected gait parameters that were used in a machine learning classification model based on support vector machine and principal component analysis. Zhou et al. (2022) collected 23 dynamic gait variables using three-dimensional (3D) accelerometer data and used random forest and artificial neural network to classify cognitive impairment.

The purpose of this study was to develop a machine vision-based gait identification method for geriatric diseases without using contact sensors or indexes, and to explore its potential as a cognitive impairment screening tool that is convenient, objective, rapid, and non-contact. To this end, a series of hyperparameters in machine vision networks for gait feature extraction and identification were deeply optimized to produce a method called Deep Optimized GaitPart (DO-GaitPart), and the optimized components and DO-GaitPart were evaluated. The performance for dementia and mild cognitive impairment (MCI) evaluation was evaluated by receiver operating characteristic/area under the curve (ROCAUC). These methods may be suitable for community screening and generalize to any gait-related approach to disease identification.

## 2 Methods

### 2.1 Participants

The current research was a cross-sectional designed analysis that included collecting part of baseline data in the West China Health and Aging Trend study, an observational study designed to evaluate factors associated with healthy aging among community-dwelling adults aged 50 years and older in western China. In 2019, we included a subset of 158 participants in Sichuan province. All participants (or their proxy respondents) were recruited by convenience and provided written informed consent to the researchers, and our institutional ethics review boards approved the study. All researchers followed the local law and protocol to protect the rights of privacy and likeness and other interests of participants in this study.

### 2.2 Definition of cognitive impairment

The Short Portable Mental Status Questionnaire (SPMSQ), a widely employed cognitive assessment tool that encompasses location, character orientation, and calculation, was applied. The established cutoff point for differentiating between healthy participants and those with mild to more severe cognitive impairment was set at a level of exceeding 3 errors in 10 questions (Pfeiffer, 1975).

### 2.3 Recording of walking video

The set of recordings was similar to that used in our previous research (Liu et al., 2021). Gait videos were shot in spacious, warm, level, well-lit indoor environments. A complete recording of each participant included six 4 m walking sequences, with three synchronized video segments shot using three different cameras (*F* = 4 mm, DS-IPC-B12V2-I, Hikvison, Zhejiang, China) for each sequence. The height from ground to cameras was approximately 1.3 m, and their angles were adjusted to ensure that the participant’s whole body could be filmed for the entire gait process between benchmarks. Data were stored by the recorder (DS-7816N-R2/8P, Hikvison, Zhejiang, China) in MP4 format at 1080p resolution.

### 2.4 Pretreatment of recording footage and data set

**Figure 1:**
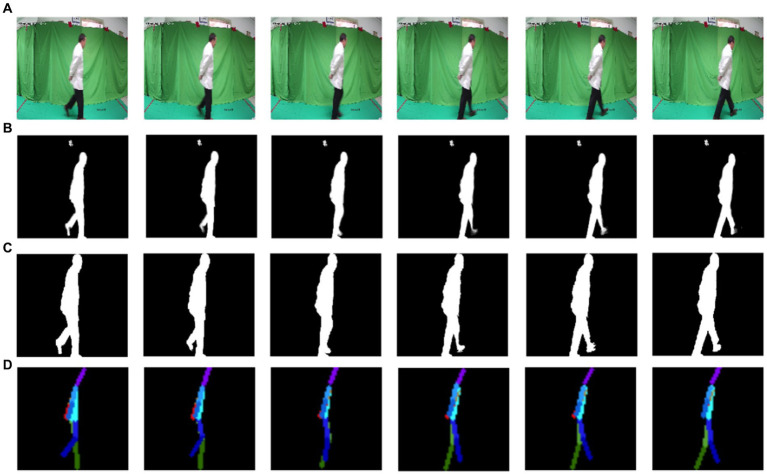
Flow of pretreatment: **(A)** static image sequence, **(B)** raw silhouette sequence, **(C)** gait silhouette sequence, and **(D)** gait skeleton sequence.

Then video files of each walking sequence were converted into static image frames (Figure 1A). The raw silhouette of walking participants was obtained through the RobustVideoMatting method (Figure 1B) (Lin et al., 2022). The FindContours function of the OpenCV library in Python was used to segment the minimum external rectangle of the maximum silhouette for the more refined silhouettes, after the participant image was centralized and normalized to 256 × 256, the gait silhouette sequence was generated (Figure 1C). The measure for spatial information extraction of skeleton points from the gait silhouette sequence was HRNet (Figure 1D) (Sun et al., 2019). Our dataset, named West China Hospital Elderly Gait (WCHEG), was used to validate the model along with two open gait video databases: CASIA-B and Gait3D. CASIA-B (Yu et al., 2006), includes data from 124 participants, with 6 normal walking sequences, 2 long clothing sequences, and 2 backpacking sequences per participant. Gait3D (Zhu et al., 2021) includes a large-scale outdoor dataset of 5,000 participants, with 1,090 total hours of gait video. The WCHEG dataset was used to test the effectiveness of the model in recognizing cognitive impairment. Each dataset uses gait skeleton images and silhouette images as model inputs, both of which have a size of 128 × 128.

### 2.5 Machine vision approach and analysis

**Figure 2:**
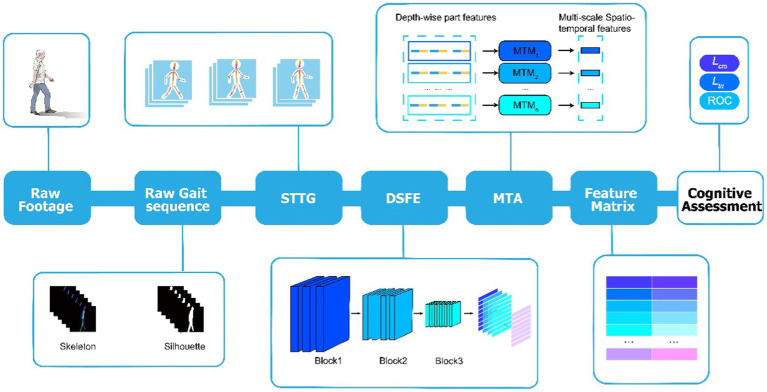
Overview of proposed gait analysis model. Extract the original gait sequence from the raw gait footage, which includes silhouette and skeleton gait images. Then, input the gait sequence into STTG to generate the template sequence, and input it into DSFE to extract depth-wise spatial features. Then, horizontally cut the output into n parts to obtain depth-wise part features. Furthermore, input each part into MTM separately to obtain the output multi-scale spatial–temporal features. Obtain the feature matrix through full connection and batch normalization, train the model through a series of loss functions such as triplet loss and cross entropy loss, and test through evaluation indicators such as ROC to achieve cognitive assessment.

Our gait dataset WCHEG included more than 400,000 frames of raw static images and corresponding silhouette and skeleton gait images. The main purpose of our optimized design was to balance computational power consumption and accuracy of the model classification. A temporal part-based module, GaitPart (Fan et al., 2020), which was designed based on the idea that the local short-range spatiotemporal features (micro-motion patterns) are the most discriminative characteristics for human gait, was applied as the original analysis work frame in the current study. To better adapt this method to the mission of cognitive impairment assessment, three novel components were designed in our analysis pipeline to achieve the proposed DO-GaitPart (Figure 2): short-term temporal template generator (STTG), depth-wise spatial feature extractor (DSFE), and multi-scale temporal aggregation (MTA).

### 2.6 STTG

**Figure 3:**
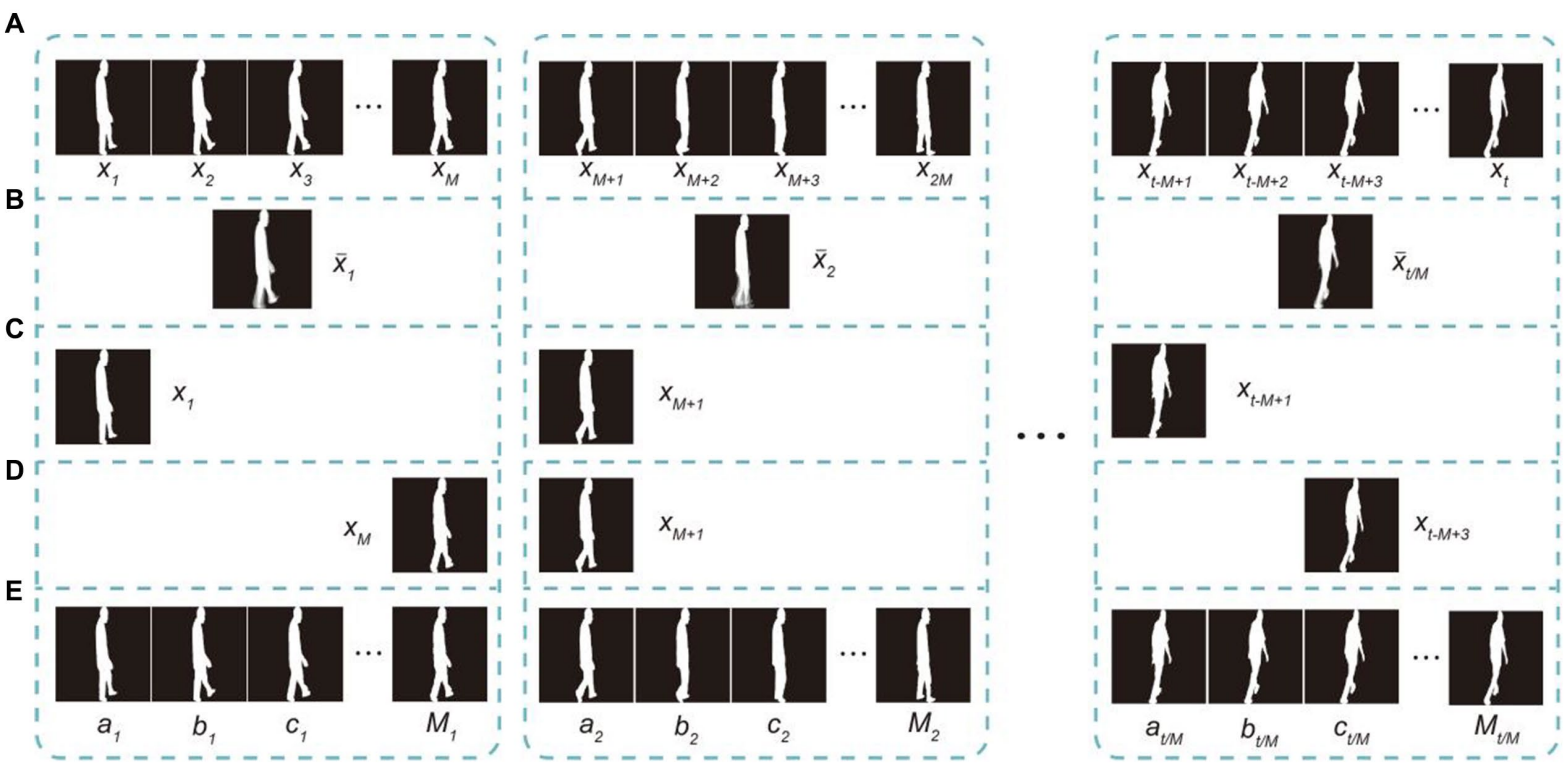
Different temporal template generating methods, with M=4 : **(A)** raw image sequence $X=\left\{x_{i}|,,,i=1|,,,2|,,,\ldots |,,,t\right\}\text{,}$ **(B)** gait energy image method, **(C)** equidistant sampling method sampling the image with equal M−1 spacers from the beginning, **(D)** simple random sampling every M images, and **(E)** short-term temporal template generator, which divides the whole gait sequence into M sets and randomly selects a set at a time.

To ensure that the input gait sequence contains a complete gait cycle with less computational cost and minimal loss of temporal information, we designed an STTG. We grouped the input dual-channel gait sequence *X*_in_ into *M* per frame and created a short-term temporal template using systematic random sampling. Most of the previous work (Fan et al., 2020; Huang X. et al., 2021; Kaur et al., 2023) directly input gait sequences into the network frame by frame, with each input gait sequence including at least one gait cycle, which meant that the sequence mean size was usually 30 frames, equivalent to more than 1 s. Because part of our participant data has the feature of cognitive impairment as well as a low stride frequency, a gait cycle often contained far more than 30 frames. As shown in Figure 3A, adjacent frames are highly similar, which generates a large amount of information redundancy and increases unnecessary computational costs. Creating the template with the gait energy image (GEI) method (Han and Bhanu, 2006), as shown in Figure 3B, leads to the loss of temporal information, because the template is based on the average of each group image. Generating the template though equidistant sampling (Figure 3C), in which fixed positions in each group are picked up and only 1/M gait images are retained in the dataset, causes a lot of waste. Generating the template using simple random sampling (Figure 3D) picks up some adjacent frames at the same time, resulting in information redundancy. STTG extracts the *k*th frame in each group, where *k* is a random value from the set $\left\{1,2,\ldots ,M\right\}$ with the equivalent probability, and in every training epoch *k* revalues (Figure 3E), which can avoid all the disadvantages of the above methods. In the current study, we compared the situation of M=2,3,4,5 and found that the best results were achieved at M=4 .

### 2.7 DSFE

We develop a DSFE to extract both global and local fine-grained spatial features from gait images. Many previous models (Huang X. et al., 2021; Li et al., 2023) used only basic convolutional neural network (CNN) modules to extract spatial features from gait images, which leads to failure of capture all the gait details. Some networks, such as GaitPart (Fan et al., 2020) developed a component focal convolutional network (FConv) to extract part features, but then just combined those part features, and as a result ignored the connections between part features. However, the DSFE extracts partial spatial features and keeps the relation between part features. The DSFE consists of three blocks. The first block contains one two-dimensional convolutional network (Conv2d) layer and one depth-wise spatial Conv2d (DS-Conv2d) layer. The following two blocks contain two Conv2d layers each. The specific network structure is shown in Table 1. For the DSFE module, we compared the location and quantity of replacing Conv2d with DS-Conv2d in Block 1, Block 2, and Block 3, respectively. We found that using DS-Conv2d in the second layer of Block1 had the best performance.

**Table 1:** Detailed parameters for depth-wise part feature extractor.

Block	Layer	In C	Out C	Kernel	Dilation	Padding
Block1	Conv2d	2	32	5	1	2
	DS-Conv2d	32	32	3	2	1
	MaxPool2d, kernel size = 2, stride = 2					
Block2	Conv2d	32	64	3	1	1
	Conv2d	64	64	3	1	1
	MaxPool2d, kernel size = 2, stride = 2					
Block3	Conv2d	64	192	3	1	1
	Conv2d	192	192	3	1	1
	MaxPool2d, kernel size = 2, stride = 2					

Conv2d, two-dimensional convolutional network; In C, input channels; Out C, output channels; kernel, kernel size; dilation, dilation rate; padding, zero padding.

**Figure 4:**
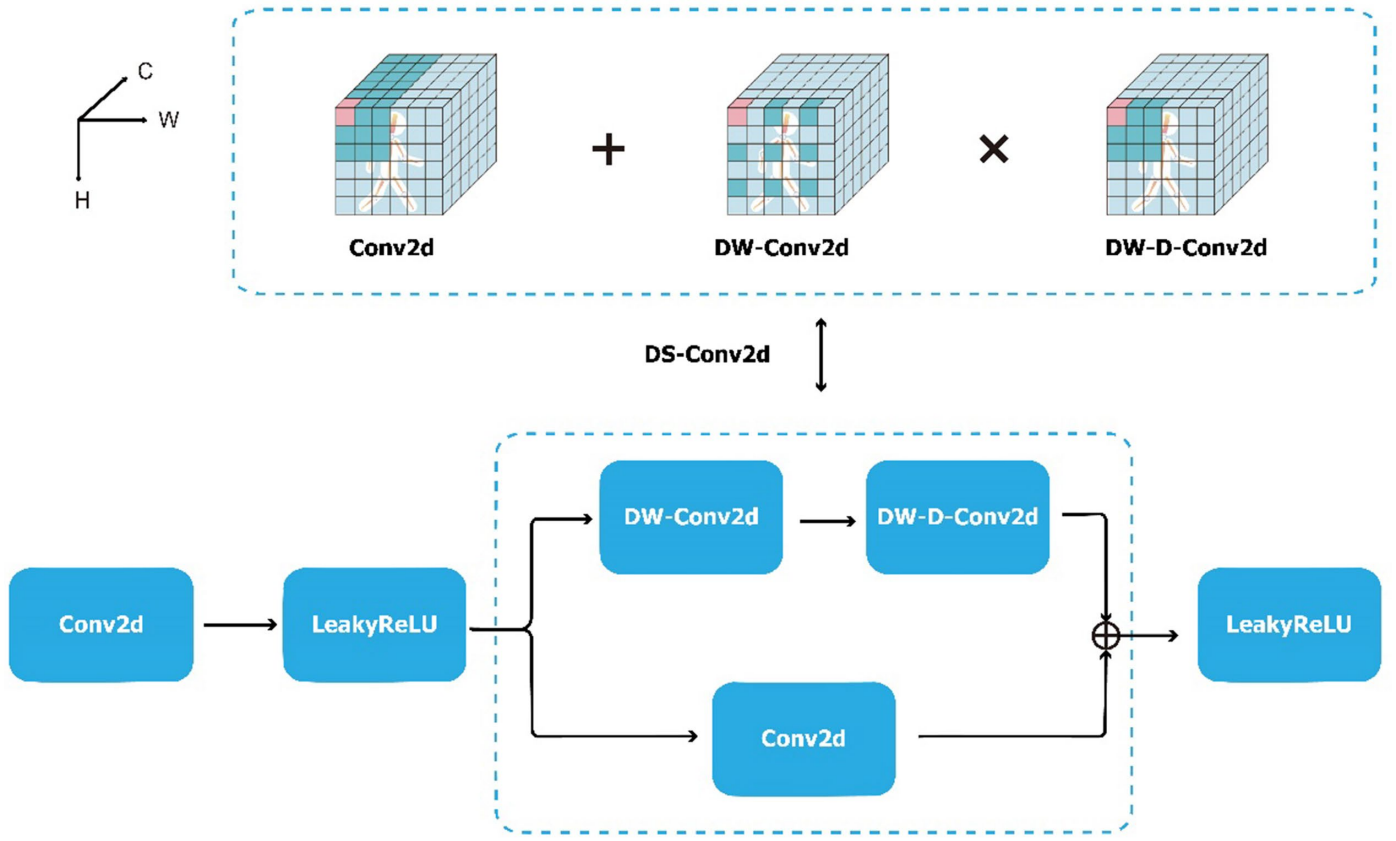
The convolution part of Block 1 in frame of DSFE, including Conv2d, DS-Conv2d and LeakyReLU. The DS-Conv2d’s convolution operation process of a pixel (pink cube) of a three-dimensional feature map of a single frame (the whole cube). The information (all color cubes) contained in the receptive field is weighted and aggregated into the pink cube. The H, W, and C of cube represent the height, width, and channel dimensions of the feature map. The dark cubes indicate the position of the convolution kernel. The convolution core size of Conv2d, DW-Conv2d, and DW-D-Conv2d are all 3 × 3, and the dilation rate of DW-Conv2d is 2. Note: The operation process has omitted the zero filling.

The structure of the DS-Conv2d module is shown in Figure 4 and can be expressed as Equation (1):


(1) $$\begin{array}{l} DS-\mathrm{Conv}2d\left(\cdot \right)=\mathrm{Conv}2d\left(\cdot \right)⊕DW-D-\\ \;\mathrm{Conv}2d\left(DW-\mathrm{Conv}2d\left(\cdot \right)\right) \end{array}$$

where depth-wise two-dimensional convolutional network (DW-Conv2d) represents depth-wise convolution (Guo et al., 2023). As shown in Figure 4, depth-wise convolution is the extraction of local features from a single-channel spatial feature map. Each convolutional kernel only performs convolution operations on a single channel. Depth-wise dilated two-dimensional convolutional network (DW-D-Conv2d) is a special type of depth-wise convolution that introduces dilated convolution to increase the model’s receptive field and extract long-range features from a single spatial feature map. The combination of the two parts takes into account local contextual information, enlarges the receptive field, and enables the extraction of richer spatial information from the gait sequence. Leaky rectified linear unit (LeakyReLU) is the activation function, which can be expressed as Equation (2):


(2) $$\mathrm{LeakyReLU}\left(x\right)=\left\{\begin{array}{l} x,x\geq 0\\ \mathrm{ax},x<0,0<a<1 \end{array}\right\}$$

### 2.8 MTA

**Figure 5:**
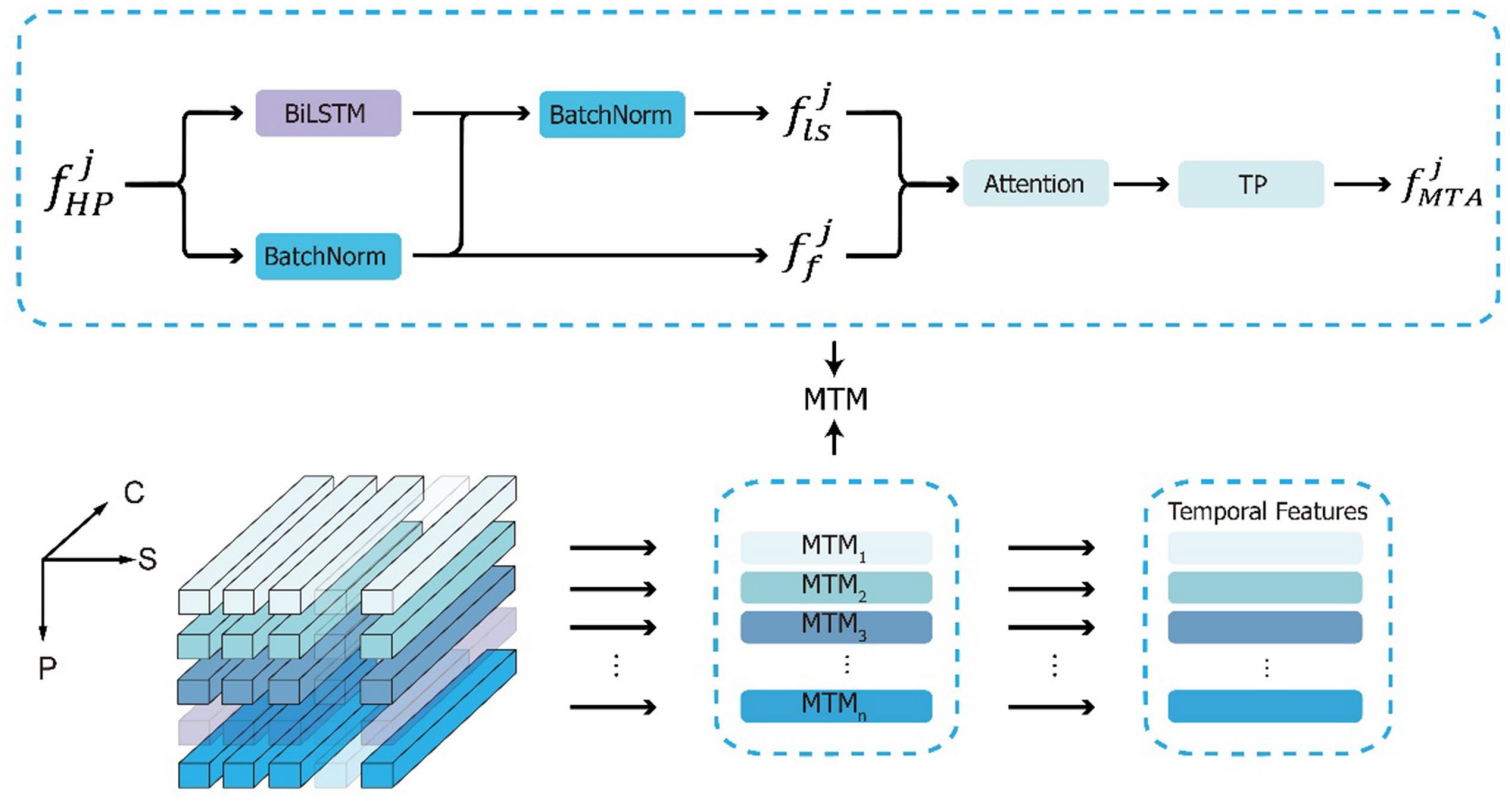
The calculation process of MTA and the details of MTM. The input is the three-dimensional gait feature maps, where P represents the component dimension, S represents the time dimension, C represents the channel dimension, and a semi transparent cube represents the omission of the feature maps. Along the component dimensions, input $F_{HP}$ into the MTMs module to obtain multi-scale time features.

MTA is composed of multiple parallel multi-scale temporal modules (MTMs), each of which is responsible for extracting features from the corresponding part of the gait sequence, acquiring multi-scale temporal features. The input to the DSFE module passes through the horizontal pooling (HP) module to obtain $F_{HP}\in R^{\frac{t}{M}\times c_{1}\times p}$ , expressed as $F_{HP}=\left\{f_{HP}^{j}|,,,j=1|,,,2|,,,\ldots |,,,p\right\}$ , where $f_{HP}^{j}\in R^{\frac{t}{M}\times c_{1}}$ represents the temporal features of the j th horizontal part. Then, the part is input into the MTM, as shown in Figure 5, extracting both frame-level $F_{f}=\left\{f_{f}^{j}|,,,j=1|,,,2|,,,\ldots |,,,p\right\}$ and long short-term temporal features $F_{ls}=\left\{f_{ls}^{j}|,,,j=1|,,,2|,,,\ldots |,,,p\right\}$ , which are then aggregated into multi-scale temporal features $F_{MTA}=\left\{f_{MTA}^{j}|,,,j=1|,,,2|,,,\ldots |,,,p\right\}$ , expressed as Equation (3):


(3) $$\begin{array}{l} f_{f}^{j}=\mathrm{BatchNorm}\left(f_{HP}^{j}\right)\\ f_{ls}^{j}=\mathrm{BatchNorm}\left(f_{f}^{j}+\mathrm{BiLSTM}\left(f_{HP}^{j}\right)\right)\\ f_{MTA}^{j}=TP\left(\mathrm{Attention}\left(\mathrm{Concat}\left(f_{f}^{j},\;f_{ls}^{j}\right)\right)\right)\\ \begin{array}{l} \mathrm{Attention}\left(\cdot \right)=\mathrm{LeakyReLU}\\ \;\left(\mathrm{Conv}1d\left(\mathrm{Dropout}\left(\begin{array}{l} \mathrm{LeakyReLU}\\ \left(\mathrm{Conv}1d\left(\cdot \right)\right) \end{array}\right)\right)\right) \end{array} \end{array}$$

where $f_{f}^{j}$ , $f_{ls}^{j}$ and $f_{MTA}^{j}$ represent the frame-level time characteristics, long short-term time features, and multi-scale time characteristics of the j th horizontal part, respectively, and for now $F_{f},F_{ls},F_{MTA}\in R^{c_{2}\times p}$ . $\mathrm{BatchNorm}\left(\cdot \right)$ indicates normalized data to mean of 0 and standard deviation of 1 by Batch. $\mathrm{BiLSTM}\left(\cdot \right)$ is a special type of long short-term memory (LSTM) (Hochreiter and Schmidhuber, 1997) known as bi-directional LSTM (BiLSTM), which is capable of accessing both past and future information in a time series, introducing more contextual dependencies and performing well in extracting short-term and long-term relationships. $\mathrm{Concat}\left(\cdot \right)$ represents the concatenation operation, connecting the frame-level time feature $F_{f}$ with the long short-term feature $F_{ls}$ along the channel dimension. $\mathrm{Attention}\left(\cdot \right)$ represents using the attention mechanism of SENet (Hu et al., 2018), which introduces the attention mechanism to focus on the relationships between channels and performs feature weighting on the channel dimension; the greater the weight equivalent the higher the correlation between the channel and key temporal information. Meanwhile, we introduce the Dropout (Srivastava et al., 2014) technique in $\mathrm{Attention}\left(\cdot \right)$ , which can mitigate the overfitting phenomenon and enhance the model’s ability to generalize to new data. $TP\left(\cdot \right)$ represents temporal pooling, and according to previous research (Fan et al., 2020), selecting $TP\left(\cdot \right)=\max\left(\cdot \right)$ yields better results.

We compared the classification results of frame-level feature, long short-term feature, and multi-scale aggregated feature. We found that long short-term feature performed better than frame level feature and multi-scale aggregated feature achieved the best classification results. By extracting frame-level and long short-term temporal features, it captures abstract features at different scale levels in the gait sequence, and then uses an attention mechanism to aggregate more distinctive temporal information.

### 2.9 Loss function and sample

During the training stage, both the separate batch all (BA+) triplet loss (Hermans et al., 2017) and the label smoothing cross entropy loss (Szegedy et al., 2016) were used to achieve more effective training results. The multiply loss function $L_{mul}$ can be defined as $L_{mul}=\lambda_{tri}L_{tri}+\lambda_{cro}L_{cro}$ , where $L_{tri}$ and $L_{cro}$ represent the BA+ triplet loss and the label smoothing cross entropy loss, respectively. $\lambda_{tri}$ and $\lambda_{cro}$ represent the weight coefficients of the loss functions. Here, $\lambda_{tri}=1.0$ = and $\lambda_{cro}=0.2$ . The batch size was set to $\left(p,k\right)=\left(4,6\right)$ , which represents that every batch includes p participants, and k gait image sequences will be picked up in every participant’s footage. The length of the analyzing sequence is 80 frames. If the length of the original sequence is less than 15 frames, it is discarded; if the length is between 15 and 80 frames, it is repeatedly sampled.

### 2.10 Comparison, ablation, and classification

CASIA-B and Gait3D was used in the comparison of individual recognition accuracy among previous gait analysis methods and DO-GaitPart. To determine which component in our model led to better adaptation for the gait analysis mission, components were removed from the total pipeline in a process known as ablation. We set eight groups of different hyperparameters for experiments and compared accuracy with that of GaitPart (composed of three Block + HP + temporal pooling modules, where each layer includes two convolutional layers and one maximum pooling layer), as baseline, in the individual recognition task. A two-class classification for mild or worse cognitive impairment gait and healthy gait features was designed to evaluate the performance of models as cognitive classifiers for the WCHEG dataset. The ground truth state for all gait features in this experiment was labelled using a previously performed SPMSQ assessment.

## 3 Results

The hardware environment is CPU, Intel i7-8700, 3.20 GHz, GPU, GeForce RTX 2080 Ti + GeForce RTX 1080 Ti. And the software development environment is Python 3.7.1, Pytorch 1.8.1.

### 3.1 Ablation study

We found that each component of our model is essential, and the addition of each component provides a positive gain in the identification results of both datasets. The best performance of the model was achieved when the three components were deployed simultaneously (Table 2). Furthermore, we conduct ablation studies on specific parameters of each module.

**Table 2:** Accuracy comparison (%) with different addition of the three components of our model on CASIA-B and WCHEG.

Group	STTG	DSFE	MTA	CASIA-B			WCHEG
				NM	BG	CL	
A	x	x	x	97.4	92.8	74.9	74.9
B	✓	x	x	97.6	93.1	76.2	76.5
C	x	✓	x	97.4	92.9	77.7	78.8
D	x	x	✓	97.8	93.2	80.3	77.7
E	✓	✓	x	97.9	93.4	79.9	79.1
F	x	✓	✓	98.0	94.7	82.8	78.2
G	✓	x	✓	97.8	94.1	83.5	77.3
H	✓	✓	✓	**98.1**	**95.4**	**84.6**	**82.5**

NM, normal walking; BG, carrying bags; CL, wearing coats or jackets. Bold values mean best performance method, model, module or algorithm in comparison.

#### 3.1.1 Analysis of different *M* numbers of STTG

The ablation experiments were designed to demonstrate the most appropriate choice of parameters for the STTG (Table 3), where the inter-frame similarity of the gait sequence decreases as the value of *M* increases, and the same number of frames can contain more gait information, reaching an optimum at *M* = 4. Whereas, when the value of M is too large, it leads to a decrease in the continuity between frames and affects the learning of the complete action of the gait. Meanwhile in the WCHEG dataset, the introduction of STTG shows a more significant performance improvement because STTG allows the input to contain more complete gait cycles.

**Table 3:** Accuracy comparison (%) with different *M* numbers of STTG on CASIA-B and WCHEG.

*M*	CASIA-B			WCHEG
	NM	BG	CL	
2	97.8	94.9	83.4	79.9
3	97.5	95.1	84.4	80.1
**4**	**98.1**	**95.4**	**84.6**	**82.5**
5	97.6	95.0	84.0	78.9

Bold values mean best performance method, model, module or algorithm in comparison.

#### 3.1.2 Analysis of different insertion positions of DS-Conv in DSFE

We conducted the ablation study by replacing the second Conv layer with DS-Conv in three different Blocks of DSFE, respectively, (Table 4). By comparison, it can be found that adding DS-Conv in Block1 has the best performance, because no pooling operation has been performed at this time, which can avoid the effects of input distortion and information loss, and better fuse contextual information and large receptive field information. Meanwhile, too much use of this module can lead to the loss of fine-grained information, which in turn leads to poorer model performance.

**Table 4:** Accuracy comparison (%) with replacing Conv with DS-Conv in different blocks of DSFE on CASIA-B and WCHEG.

Block 1	Block 2	Block 3	CASIA-B			WCHEG
			NM	BG	CL	
✓	x	x	**98.1**	**95.4**	**84.6**	82.5
✓	✓	x	97.5	93.9	83.1	78.1
✓	✓	✓	97.1	94.3	83.7	79.2
x	✓	x	96.8	94.0	83.2	78.2
x	x	✓	95.2	89.1	72.6	71.1

Bold values mean best performance method, model, module or algorithm in comparison.

#### 3.1.3 Effectiveness of MTA

In order to validate the effectiveness of MTA, we set up ablation experiments (Table 5). It can be found that BiLSTM will obtain better results compared to LSTM for extracting long and short-term features, because BiLSTM has the characteristic of bidirectional computation, which acquires more comprehensive temporal features. Meanwhile, the use of Attention better fuses the multi-scale features and reduces the risk of overfitting by dropout method.

**Table 5:** Accuracy comparison (%) with different algorithms used by MTA on CASIA-B and WCHEG.

$F_{f}$	F1s	Attention	CASIA-B			WCHEG
			NM	BG	CL	
x	LSTM	x	97.5	93.1	79.2	78.9
✓	LSTM	✓	97.8	94.2	82.8	81.1
x	BiLSTM	x	97.7	93.7	81.2	81.3
✓	BiLSTM	✓	**98.1**	**95.4**	**84.6**	**82.5**

Bold values mean best performance method, model, module or algorithm in comparison.

### 3.2 Comparison in gait identification task

As shown in Table 6, the accuracy of the proposed method on CASIA-B dataset was compared with several previous gait identification methods, including GaitSet (Chao et al., 2019), GaitPart (Fan et al., 2020), MT3D (Lin et al., 2020), 3D Local (Huang Z. et al., 2021), TransGait (Han and Bhanu, 2006), CSTL (Lin et al., 2020), GLN (Huang Z. et al., 2021), GaitGL (Liang et al., 2022), SMPLGait (Zhu et al., 2021). The results show that DO-GaitPart has excellent gait recognition on the CASIA-B dataset, and is superior to the comparison methods in the BG walking scene. Meanwhile, DO-GaitPart has the best performance on the Gait3D dataset compared to the comparison methods.

**Table 6:** Accuracy in comparison with previous gait identification methods on CASIA-B and Gait3D.

Method	CASIA-B			Gait3D			
	NM (%)	BG (%)	CL (%)	R-1 (%)	R-5 (%)	mAP (%)	mINP
GaitSet (Hermans et al., 2017)	95.0	87.2	70.4	42.6	63.1	33.7	19.7
GaitPart (Fan et al., 2020)	96.2	91.5	78.7	29.9	50.6	23.3	13.2
MT3D (Szegedy et al., 2016)	96.7	93.1	81.5	—	—	—	—
3D Local (Chao et al., 2019)	97.5	94.3	83.7	—	—	—	—
TransGait (Han and Bhanu, 2006)	98.1	94.9	85.8	—	—	—	—
CSTL (Lin et al., 2020)	**98.7**	94.8	**88.7**	12.2	21.7	6.44	3.28
GLN (Huang et al., 2021)	96.9	94.0	77.5	42.2	64.5	33.1	19.6
GaitGL (Liang et al., 2022)	97.4	94.5	83.6	23.5	38.5	16.4	9.2
SMPLGait w/o 3D (Zhu et al., 2021)	—	—	—	47.7	67.2	37.6	22.2
DO-GaitPart	98.1	**95.4**	84.6	**49.2**	**68.2**	**39.1**	**24.1**

NM, normal walking; BG, carrying bags; CL, wearing coats or jackets. Bold values mean best performance method, model, module or algorithm in comparison.

### 3.3 Characterization of participants

We compared the background information of participants between the training/validation and test sets (Table 7). We found no significant differences in age, gender, education level, or cognitive status prevalence between the training/validation and test sets.

**Table 7:** Characterization and cognitive status of participants among 158 older adults.

Characteristic	Prevalence, *n* (%)	Within cognitive status, *n* (%)		Within analysis set, n (%)	
		Healthy	MCI or Worse	Training and validation	Test
All participants	158 (100.0)	66 (41.1)	92 (58.3)	110 (69.6)	48 (30.4)
Age, years (Mean ± SD)	65.4 ± 8.2	64.5 ± 1.0	66.0 ± 0.9	65.5 ± 0.8	65.3 ± 1.2
**Gender**					
Male	30 (19.0)	18 (27.3)	12 (13.0)	20 (18.2)	10 (20.8)
Female	128 (81.0)	48 (72.7)	80 (87.0)	90 (81.8)	38 (79.2)
**Cognitive status**					
Healthy	66 (41.8)	—	—	46 (41.8)	20 (41.7)
MCI or Worse	92 (58.2)	—	—	64 (58.2)	28 (58.3)
**Education level**					
Primary or illiterate	130 (82.3)	39 (59.1)	91 (98.9)	91 (82.7)	39 (81.3)
Junior high	22 (13.9)	21 (31.8)	1 (1.1)	14 (12.7)	8 (16.7)
Senior high or higher	6 (3.8)	6 (9.1)	0 (0.0)	5 (4.5)	1 (2.1)
**Marital status**					
Married	130 (82.3)	55 (83.3)	75 (81.5)	91 (82.7)	39 (81.3)
Others	28 (17.7)	11 (16.7)	17 (18.5)	19 (17.3)	9 (18.8)
**Body and cognitive measurement (mean ± SD)**					
BMI, kg/m^2^	25.5 ± 3.7	25.2 ± 0.4	25.8 ± 0.4	25.6 ± 0.4	25.5 ± 0.5
Time of 4 m walking, s	7.2 ± 2.0	6.6 ± 0.2	7.7 ± 0.2	7.2 ± 0.2	7.2 ± 0.2
Wrong answers in SPMSQ	2.9 ± 2.0	0.9 ± 0.1	4.3 ± 0.1	2.9 ± 0.2	2.8 ± 0.3

MCI, mild cognitive impairment; BMI, body mass index.

### 3.4 Classification

**Figure 6:**
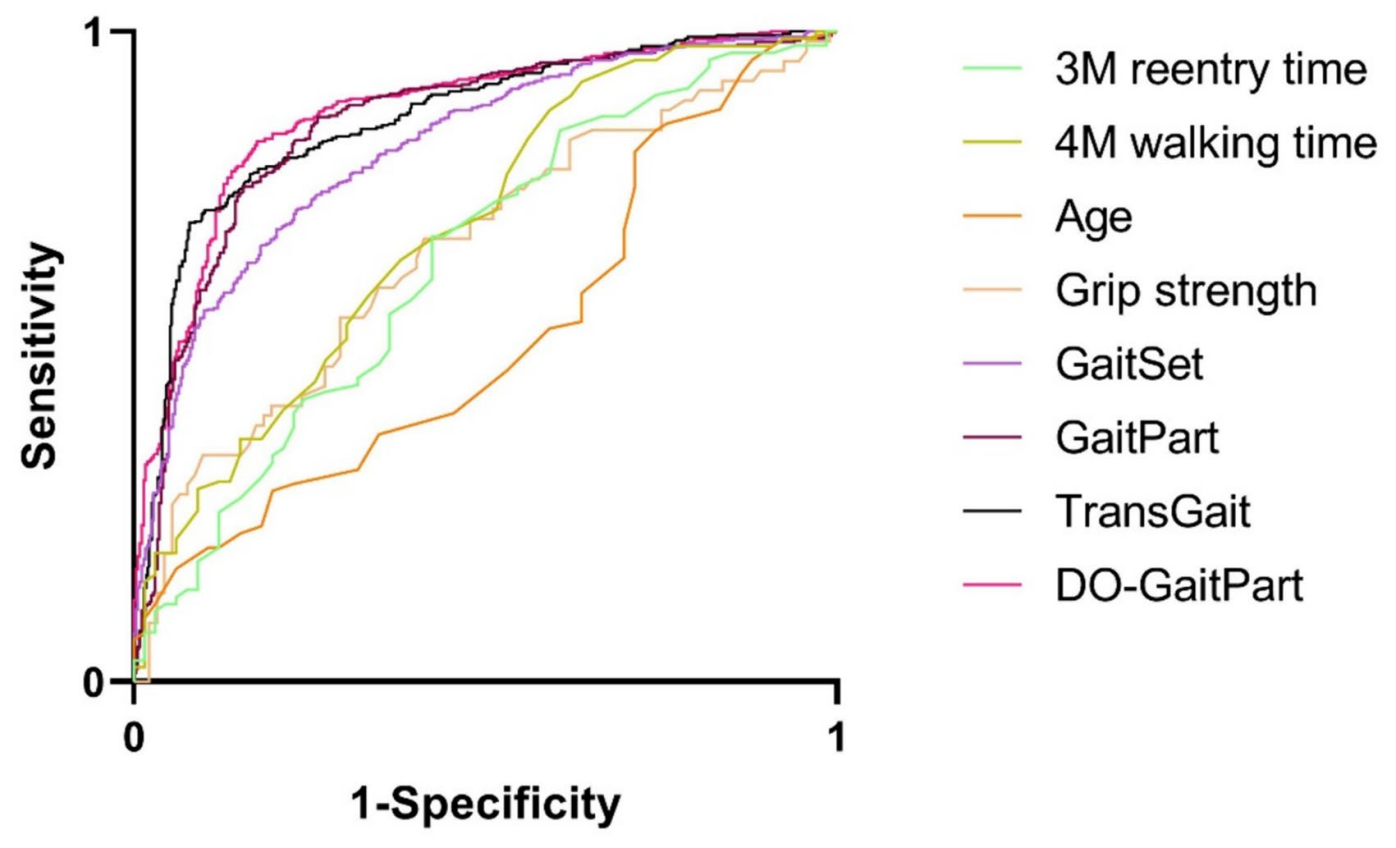
Receiver operating characteristic/area under the curve (ROCAUC) of test set via DO-GaitPart, GaitPart, GaitSet, Grip strength, age, 4M walking time, 3M reentry time.

Table 8 presents a comparison of predictive performance among various methods for cognitive state classification, with a focus on gait features. Machine vision-based classification techniques, specifically DO-GaitPart, GaitSet, and GaitPart, exhibit notably superior performance when compared to approaches considering age, grip strength, and walking time characteristics. The significance levels for all methods, except for age and 3 m re-entry time, are less than 0.001, providing statistical evidence for the potential of these methods in identifying cognitive impairment. Notably, among these gait-based methods, DO-GaitPart achieves the highest ROCAUC value (0.876, Figure 6) with a 95% confidence interval of 0.852–0.900, indicating its robust predictive capability for cognitive impairment. This performance stands significantly ahead of other methods, as evidenced by the substantially lower significance values. Moreover, DO-GaitPart operates with remarkable efficiency, consuming a mere 0.013 s per gait sequence, ensuring swift response to gait-related information. Conversely, methods relying on age and grip strength exhibit comparatively lower ROCAUC values, signaling their limited effectiveness in cognitive state classification. In summary, these result underscores the efficacy of machine vision-based gait feature classification methods, particularly highlighting DO-GaitPart, in predicting cognitive impairment.

**Table 8:** Predictive performance of cognitive state classification via different method.

Method	ROCAUC (0.95 confidence)	Significance	Gink coefficient	Max K-S
GaitSet (Hermans et al., 2017)	0.821 (0.793–0.849)	<0.001	0.642	0.496
GaitPart (Zhu et al., 2021)	0.850 (0.824–0.875)	<0.001	0.699	0.581
TransGait (Han and Bhanu, 2006)	0.864 (0.839–0.890)	<0.001	0.729	0.626
**DO-GaitPart**	**0.876 (0.852–0.900)**	**<0.001**	**0.752**	**0.656**
Age	0.531 (0.429–0.623)	0.508	0.062	0.113
Grip strength	0.663 (0.576–0.750)	<0.001	0.327	0.269
4 m walking time	0.696 (0.613–0.779)	<0.001	0.392	0.288
3 m re-entry time	0.646 (0.558–0.734)	0.001	0.292	0.261

ROCAUC, receiver operating characteristic/area under the curve. Bold values mean best performance method, model, module or algorithm in comparison.

## 4 Discussion

In the current study, a machine vision method based on visible light camera footage of walking was implemented to identify mild and worse cognitive impairment among older adults. First, walking video dataset labelled using a cutoff of three errors on the SPMSQ consisting of 158 participants aged 50 and older was created. All images of gait sequences were segmented, normalized, and refined. Skeleton point information was extracted from sequences by HRNet application. Gait skeleton points and silhouette information were used in a trained recognition network, DO-GaitPart. To decrease computational cost and minimize the loss of time information, STTG was applied in the template generation stage. DSFE was used to extract more spatial features and keep the relation between features. Attention mechanism-based MTA extracted more multi-scale temporal features, including frame-level and long short-term temporal features, and aggregated more characteristic features.

After training, machine vision methods achieved better predictive performance globally than age, grip strength, or 4 m walking time in the healthy and cognitive impairment classification task. Although silhouettes contain information regarding variation in walking appearance and movement, long clothing and carrying a backpack could mislead the feature extrication process in silhouette-only methods. Here, both skeleton points and silhouette information were used to generate gait features, as skeleton points characterize human joint movement and decrease the impact of clothing and carried objects. The data input into the analysis model should contain a full gait cycle, which has a large computational cost. Compared with the previous sampling method, random sampling, STTG greatly increases the information entropy that the input sequence contains and maintains the same computational cost. GaitPart developed FConv to extract part features, but it ignored the connection between part features. With the applied depth-wise dilation convolution and depth-wise dilation convolution, DSFE comprehensively extracted contextual information and long-range features. GaitPart considered long-range features to have little effect, and provided a micro-motion capture module to extract short-range features. In our experiments, long-range features also have unique advantages in gait recognition, compared with short-range features. Therefore, we design an MTA module to aggregate multi-scale temporal features, including frame-level features, short-term features, and long-term features. Although DO-GaitPart exhibited good performance in cognitive identification task, long clothing that covered the participant’s body could decrease the precision of skeleton point identification and segmentation, thus influencing the performance of the overall method. Like most nonlinear regression algorithms, part of the analysis process in the current study was not interpretable, understandable, and straightforward (Liang et al., 2022).

Research on cognitive MCI and Alzheimer’s disease increasingly emphasizes the application of machine vision and modal fusion algorithms. Key techniques, including prior-guided adversarial learning, brain structure–function fusion, and multimodal representation learning, are being actively explored to improve diagnostic precision and enable earlier predictions of cognitive decline (Zuo et al., 2021, 2023, 2024). As these techniques evolve, they are poised to significantly advance our comprehension and treatment of neurodegenerative conditions. However, its performance in cognitive impairment classification tasks is still limited by the dataset size and the uncertainty of cognitive impairment labels. In future work, expanding the dataset and incorporating additional cognitive function screening scales, such as MMSE and MoCA, will ensure more accurate and stable data labeling. Additionally, the analysis of gait features should be extended to improve the model’s ability to recognize different levels of cognitive impairment.

## 5 Conclusion

This study introduces DO-GaitPart, a machine vision method for identifying cognitive impairment in the elderly from walking videos, featuring three key advancements: STTG, DSFE, and MTA. Addressing the global challenge of managing progressive cognitive decline (Jia et al., 2021), this non-invasive, cost-effective tool optimizes elder healthcare by conserving manpower and broadening its scope (Newey et al., 2015; Reynolds et al., 2022). Utilizing affordable cameras, it enables high-frequency, long-term cognitive assessments, potentially inspiring self-reporting tests and telemedicine for cognitive health (Charalambous et al., 2020; Hernandez et al., 2022). The method’s machine learning algorithms also show promise for detecting other geriatric conditions, enhancing the toolkit for geriatric care.

## Data availability statement

The datasets presented in this article are not readily available because their containing information that could compromise the privacy of research participants. Requests to access the datasets should be directed to YL, liuyixin@wchscu.cn.

## Ethics statement

The studies involving humans were approved by Biomedical Ethics Committee of West China Hospital, Sichuan University. The studies were conducted in accordance with the local legislation and institutional requirements. The participants provided their written informed consent to participate in this study.

## Author contributions

YQ: Methodology, Writing – original draft, Validation. HZ: Visualization, Writing – original draft. LQ: Writing – review & editing, Supervision. QL: Data curation, Writing – original draft. HJ: Funding acquisition, Writing – review & editing, Data curation. SX: Funding acquisition, Writing – review & editing, Data curation. YL: Conceptualization, Data curation, Funding acquisition, Writing – original draft, Writing – review & editing. XH: Funding acquisition, Supervision, Validation, Writing – review & editing.

## References

[ref1] Allain H. Akwa Y. Lacomblez L. Lieury A. Bentue-Ferrer D. (2007). Impaired cognition and attention in adults: pharmacological management strategies. Neuropsychiatr. Dis. Treat. 3, 103–116. doi: 10.2147/nedt.2007.3.1.103, PMID: 19300541 PMC2654526

[ref2] Chao H. He Y. Zhang J. Feng J. (2019). GaitSet: regarding gait as a set for cross-view gait recognition. 33rd AAAI Conference on Artificial Intelligence/31st Innovative Applications of Artificial Intelligence Conference/9th AAAI Symposium on Educational Advances in Artificial Intelligence. Honolulu, HI. 8126–8133.

[ref3] Charalambous A. P. Pye A. Yeung W. K. Leroi I. Neil M. Thodi C.. (2020). Tools for app-and web-based self-testing of cognitive impairment: systematic search and evaluation. J. Med. Internet Res. 22:e14551. doi: 10.2196/14551, PMID: 31951218 PMC6996724

[ref4] Chen P. H. Lien C. W. Wu W. C. Lee L. S. Shaw J. S. (2020). Gait-based machine learning for classifying patients with different types of mild cognitive impairment. J. Med. Syst. 44:107. doi: 10.1007/s10916-020-01578-7, PMID: 32328889

[ref5] Fan C. Peng Y. Cao C. Liu X. Hou S. Chi J.. (2020). GaitPart: temporal part-based model for gait recognition. 2020 IEEE/CVF Conference on Computer Vision and Pattern Recognition (CVPR). Seattle, WA. 14213–14221.

[ref6] Guo M.-H. Lu C.-Z. Liu Z.-N. Cheng M.-M. Hu S. M. (2023). Visual attention network. Comput. Visual Media 9, 733–752. doi: 10.1007/s41095-023-0364-2

[ref7] Hackett R. A. Davies-Kershaw H. Cadar D. Orrell M. Steptoe A. (2018). Walking speed, cognitive function, and dementia risk in the English longitudinal study of ageing. J. Am. Geriatr. Soc. 66, 1670–1675. doi: 10.1111/jgs.15312, PMID: 29508385 PMC6127007

[ref8] Han J. Bhanu B. (2006). Individual recognition using gait energy image. IEEE Trans. Pattern Anal. Mach. Intell. 28, 316–322. doi: 10.1109/TPAMI.2006.38, PMID: 16468626

[ref9] Hermans A. Beyer A. L. Leibe B. (2017). In defense of the triplet loss for person re-identification. *arXiv*. Available at: 10.48550/arXiv.1703.07737. [Epub ahead of preprint]

[ref10] Hernandez H. H. C. Ong P. L. Anthony P. Ang S. L. Salim N. B. M. Yew P. Y. S.. (2022). Cognitive assessment by telemedicine: reliability and agreement between face-to-face and remote videoconference-based cognitive tests in older adults attending a memory clinic. Ann. Geriatr. Med. Res. 26, 42–48. doi: 10.4235/agmr.22.0005, PMID: 35236016 PMC8984169

[ref11] Hochreiter S. Schmidhuber J. (1997). Long short-term memory. Neural Comput. 9, 1735–1780. doi: 10.1162/neco.1997.9.8.1735 9377276

[ref12] Horst F. Lapuschkin S. Samek W. Muller K. R. Schollhorn W. I. (2019). Explaining the unique nature of individual gait patterns with deep learning. Sci. Rep. 9:2391. doi: 10.1038/s41598-019-38748-8, PMID: 30787319 PMC6382912

[ref13] Hu J. Shen L. Sun G. (2018). Squeeze-and-excitation networks. 31st IEEE/CVF Conference on Computer Vision and Pattern Recognition (CVPR). Salt Lake City, UT. 7132–7141.

[ref14] Huang Z. Xue D. X. Shen X. Tian X. M. Li H. Q. Huang J. Q.. (2021). 3D local convolutional neural networks for gait recognition. 18th IEEE/CVF International Conference on Computer Vision (ICCV). 14900–14909.

[ref15] Huang X. Zhu D. Wang H. Wang X. Yang B. He B.. (2021). Context-sensitive temporal feature learning for gait recognition. 18th IEEE/CVF International Conference on Computer Vision (ICCV), pp. 12889–12898.

[ref16] Jia J. Xu J. Liu J. Wang Y. Wang Y. Cao Y.. (2021). comprehensive management of daily living activities, behavioral and psychological symptoms, and cognitive function in patients with Alzheimer’s disease: a Chinese consensus on the comprehensive management of Alzheimer's disease. Neurosci. Bull. 37, 1025–1038. doi: 10.1007/s12264-021-00701-z, PMID: 34050523 PMC8275730

[ref17] Kaur R. Motl R. W. W. Sowers R. Hernandez M. E. E. (2023). A vision-based framework for predicting multiple sclerosis and Parkinson’s disease gait dysfunctions-a deep learning approach. IEEE J. Biomed. Health Inform. 27, 190–201. doi: 10.1109/JBHI.2022.3208077, PMID: 36126031

[ref18] Leisman G. Moustafa A. A. Shafir T. (2016). Thinking, walking, talking: integratory motor and cognitive brain function. Front. Public Health 4:94. doi: 10.3389/fpubh.2016.00094 27252937 PMC4879139

[ref19] Li G. Guo L. Zhang R. Qian J. Gao S. (2023). TransGait: multimodal-based gait recognition with set transformer. Appl. Intell. 53, 1535–1547. doi: 10.1007/s10489-022-03543-y

[ref20] Liang D. Frederick D. A. Lledo E. E. Rosenfield N. Berardi V. Linstead E.. (2022). Examining the utility of nonlinear machine learning approaches versus linear regression for predicting body image outcomes: the U.S. Body Project I. Body Image 41, 32–45. doi: 10.1016/j.bodyim.2022.01.013, PMID: 35228102

[ref21] Lin S. Yang L. Saleemi I. Sengupta S. Soc I. C. (2022). Robust high-resolution video matting with temporal guidance. 22nd IEEE/CVF Winter Conference on Applications of Computer Vision (WACV). Waikoloa, HI. 3132–3141.

[ref22] Lin B. B. Zhang S. L. Bao F. (2020). Gait recognition with multiple-temporal-scale 3D convolutional neural network. 28th ACM International Conference on Multimedia (MM). 3054–3062.

[ref23] Lisko I. Kulmala J. Annetorp M. Ngandu T. Mangialasche F. Kivipelto M. (2021). How can dementia and disability be prevented in older adults: where are we today and where are we going? J. Intern. Med. 289, 807–830. doi: 10.1111/joim.13227, PMID: 33314384 PMC8248434

[ref24] Liu Y. He X. Wang R. Teng Q. Hu R. Qing L.. (2021). Application of machine vision in classifying gait frailty among older adults. Front. Aging Neurosci. 13:757823. doi: 10.3389/fnagi.2021.757823, PMID: 34867286 PMC8637841

[ref25] Livingston G. Huntley J. Sommerlad A. Ames D. Ballard C. Banerjee S.. (2020). Dementia prevention, intervention, and care: 2020 report of the Lancet Commission. Lancet 396, 413–446. doi: 10.1016/S0140-6736(20)30367-6, PMID: 32738937 PMC7392084

[ref26] Newey S. Davidson P. Nazir S. Fairhurst G. Verdicchio F. Irvine R. J.. (2015). Limitations of recreational camera traps for wildlife management and conservation research: a practitioner’s perspective. Ambio 44, 624–635. doi: 10.1007/s13280-015-0713-1, PMID: 26508349 PMC4623860

[ref27] Ohlin J. Ahlgren A. Folkesson R. Gustafson Y. Littbrand H. Olofsson B.. (2020). The association between cognition and gait in a representative sample of very old people—the influence of dementia and walking aid use. BMC Geriatr. 20:34. doi: 10.1186/s12877-020-1433-3, PMID: 32005103 PMC6995040

[ref28] Pfeiffer E. (1975). A short portable mental status questionnaire for the assessment of organic brain deficit in elderly patients. J. Am. Geriatr. Soc. 23, 433–441. doi: 10.1111/j.1532-5415.1975.tb00927.x, PMID: 1159263

[ref29] Reynolds C. F. 3rd Jeste D. V. Sachdev P. S. Blazer D. G. (2022). Mental health care for older adults: recent advances and new directions in clinical practice and research. World Psychiatry 21, 336–363. doi: 10.1002/wps.20996, PMID: 36073714 PMC9453913

[ref30] Savica R. Wennberg A. M. Hagen C. Edwards K. Roberts R. O. Hollman J. H.. (2017). Comparison of gait parameters for predicting cognitive decline: the Mayo Clinic study of aging. J. Alzheimers Dis. 55, 559–567. doi: 10.3233/JAD-160697, PMID: 27662317 PMC5378311

[ref31] Srivastava N. Hinton G. Krizhevsky A. Sutskever I. Salakhutdinov R. (2014). Dropout: a simple way to prevent neural networks from overfitting. J. Mach. Learn. Res. 15, 1929–1958.

[ref32] Sun K. Xiao B. Liu D. Wang J. Soc I. C. (2019). Deep high-resolution representation learning for human pose estimation. 32nd IEEE/CVF Conference on Computer Vision and Pattern Recognition (CVPR). Long Beach, CA. 5686–5696.

[ref33] Szegedy C. Vanhoucke V. Ioffe S. Shlens J. Wojna Z. (2016) Rethinking the inception architecture for computer vision. 2016 IEEE Conference on Computer Vision and Pattern Recognition (CVPR). Seattle, WA. 2818–2826.

[ref34] Woodford H. J. George J. (2007). Cognitive assessment in the elderly: a review of clinical methods. QJM 100, 469–484. doi: 10.1093/qjmed/hcm051 17566006

[ref35] Yu S. Q. Tan D. L. Tan T. N. (2006). A framework for evaluating the effect of view angle, clothing and carrying condition on gait recognition. 18th International Conference on Pattern Recognition (ICPR 2006). Hong Kong. 441.

[ref36] Yun S. Ryu S. (2022). The effects of cognitive-based interventions in older adults: a systematic review and meta-analysis. Iran. J. Public Health 51, 1–11. doi: 10.18502/ijph.v51i1.8286, PMID: 35223620 PMC8837877

[ref37] Zhou Y. van Campen J. Hortobágyi T. Lamoth C. J. C. (2022). Artificial neural network to classify cognitive impairment using gait and clinical variables. Intell.-Based Med. 6:100076. doi: 10.1016/j.ibmed.2022.100076

[ref38] Zhu Z. Guo X. Yang T. Huang J. Deng J. Huang G.. (2021). Gait recognition in the wild: a benchmark. 2021 IEEE/CVF International Conference on Computer Vision (ICCV). 14769–14779.

[ref39] Zuo Q. Lei B. Shen Y. Liu Y. Feng Z. Wang S. (2021). Multimodal representations learning and adversarial hypergraph fusion for early Alzheimer’s disease prediction. Pattern Recognition and Computer Vision. Springer, Cham

[ref40] Zuo Q. Wu H. Chen C. L. P. Lei B. Wang S. (2024). Prior-guided adversarial learning with hypergraph for predicting abnormal connections in Alzheimer's disease. IEEE Trans. Cybern. 54, 3652–3665. doi: 10.1109/TCYB.2023.3344641, PMID: 38236677

[ref41] Zuo Q. Zhong N. Pan Y. Wu H. Lei B. Wang S. (2023). Brain structure-function fusing representation learning using adversarial decomposed-VAE for analyzing MCI. IEEE Trans. Neural Syst. Rehabil. Eng. 31, 4017–4028. doi: 10.1109/TNSRE.2023.3323432 10.1109/TNSRE.2023.3323432 37815971

